# Health Management Workforce for India in 2030

**DOI:** 10.3389/fpubh.2018.00227

**Published:** 2018-08-20

**Authors:** Ritika Tiwari, Himanshu Negandhi, Sanjay P. Zodpey

**Affiliations:** ^1^Symbiosis International (Deemed University) Pune, India; ^2^Indian Institute of Public Health—Delhi Public Health Foundation of India, New Delhi, India; ^3^Public Health Foundation of India New Delhi, India

**Keywords:** public health education, health management, health administration, hospital administration, public health professional, India

## Abstract

**Introduction:** Since its launch in the year 2005, National Rural Health Mission (NHM) has exhibited a felt need for health management training in India against the background of a shortfall of trained public health managers in the country. In India's context, health (hospital) management professionals are those, who are working in the health sector, belonging to medical and non-medical backgrounds and are trained in health (hospital) management/administration programs or other public health programs (for e.g., Master of Public Health) wherein health (hospital) management/administration is significant part of the curriculum. The presence of trained management professionals in the health sector has grown over the years.

**Objectives:** To estimate the supply, need and requirement for health management professionals for India in the year 2030.

**Materials and methods:** The supply data for health management professionals was calculated based on the output from various academic programs related to health management/administration and other public health programs. Need was calculated using “service target approach” and benchmark analysis with 2.97 health managers per 100,000 population (NACCHO 2011). Supply-need gap was estimated using normative need as base number for projections whereas for rest of the years (2018–2030) projections were done at a constant growth rate as per India's population projections.

**Results:** The overall supply capacity of trained health management professionals was 3,463 for 2017. However, based upon a service target approach India requires 11,304 health management professionals in 2017. If India is to reach the normative standards of 2.97 health managers per 100,000 population, the country would need 39,774 health management professionals in 2017. This need would increase to approximately 44,936 health management professionals by the year 2030 to maintain the normative standard of 2.97 health managers per 100,000 population.

**Conclusions:** The supply side will match the requirement of HMPs earliest by the year 2026 in a high seat occupancy scenario.Moreover, there is a need to improve the quality of the output in terms of an explicitly stated and standardized competency framework that is tailored to the Indian context.

## Introduction

As per the World Health Organization (WHO), a lack of leadership and management capacity is a constraint on the efficient operation of public and private health sectors despite the time and money spent by governments to strengthen these capacities (1). Effective leadership and management in the health services is the key to using the available resources effectively and achieving measurable results. A study undertaken by European Health Management Association (EHMA) supports and re-emphasizes the importance and value of health management in order to effectively address the current health systems' challenges and suggests for its further development (2). Management is a very important skill in the health sector where health managers struggle for the utilization of scarce resources.

A health management professional (HMP) is expected to juggle a variety of responsibilities. The presence of trained management professionals in the health sector, to work for hospitals, pharmaceutical companies, health insurance and third-party administration, and other healthcare provider organizations—is a growing phenomenon (3). HMPs adopt strategic approaches, describe and understand the health experience of populations and analyze the factors affecting health (4). HMPs operate in a multi-professional, multi-agency environment to achieve multisectoral changes (4). As per Sharma et al. trained health management professionals are needed across the entire health system of the country (3).

Over the years, India has made strategic investment toward management of health system; the National Rural Health Mission (NRHM)—now the National Health Mission (NHM)—was launched in the year 2005 to ensure quality and affordable healthcare for all. The National Health Mission (NHM) has also exhibited a felt need for health management training (5). Effective health management systems are crucial to the successful coordination of multiple resources (financial and human resources), diverse communities and complex processes (6). Systems leading to better management allow for effective coordination of public and private sector efforts and ensure universal health coverage. Thus, systemic reforms are needed to ensure effective functioning and delivery of health care services, in both rural and urban areas (6). In the year 2007, the Task Force on Medical Education for the NRHM has also recommended reformative and remedial action in medical education and health manpower development (7). The Post Graduate Diploma in Public Health Management (PGDPHM) program is a Government of India supported program which was launched under the NRHM to address to the growing demand in the health management capacity of the public sector (3).

HMPs working in public sector are usually from a medical background, with limited training in terms of management and administration. Currently the supply of HMPs come from two distinct streams—(i) medical colleges and (ii) other institutes.

(i)Programs offered exclusively to medical graduates through medical colleges:

1. Health management/administration; Hospital management/ administration
2. Community medicine; preventive and social medicine; public health

(ii) Programs offered to medical and non-medical graduates through institutes other than medical colleges:

1. Health management/administration
2. Hospital management/administration
3. Health and hospital management/ administration
4. Master of Public Health (wherein health (hospital) management/administration is significant part of the curriculum).

At block, district, and state levels, medical doctors are given the responsibilities to cater to the administrative issues. The doctors, nurses, paramedics, and the entire hospital staff are expected to multitask, in addition to routine clinical duties. However, the current shortage of health workers is putting strain on public health facilities (8). The specialized requirement for health management/administration is important to strengthen the systemic efficiency within the health sector. It is suggested that certain administrative responsibilities can be off-loaded from health workers delivering healthcare (9). The recently released National Health Policy (NHP) 2017 has also echoed the requirement of specialized management skills and it has proposed the creation of a public health management cadre in all states, with a qualification in public health or related discipline as an entry criterion (10). The NHP 2017 also advocates an appropriate career structure and recruitment policy to attract young and talented multidisciplinary professionals for this cadre.

There is limited information regarding the supply of HMPs available currently in the country. During the last two decades, there has been an expansion and growth in institutions offering health management programs. Various universities/institutions in the country are offering these programs in health (hospital) management/administration but there is no single point source to provide information regarding such institutions and programs. Also in the absence of a public health council or a body that is governing public health education, we are witnessing variability in public health program design, curricular contents (11), competencies acquired (11) and ultimately job proficiencies (12, 13) This manuscript reports the results of an assessment of the supply side and the need for health management professionals in the country. We provide forecasts of the number of health management professionals in India until 2030. The operational definitions for the manuscript have been provided in **Box 1**.

Box 1. **Operational Definitions for the manuscript.**

- **Supply:** Supply of trained health management professionals from Indian educational institutions.
- **Need:** The normative need for health management professionals as reported in literature.

## Materials and methods

For our current work in India, we included Medical Colleges and Institutions training HMPs in India in health (hospital) management/administration (as mentioned in Box 2). To estimate the supply of HMPs we have classified these programs into six categories.

Box 2 **Medical colleges and institutes training HMPs in India [includes health (hospital) management/administration programs]**

(i) Health (hospital) management / administration programs offered exclusively to medical graduates through medical colleges

1. Health management/ administration; Hospital management/ administration: MD - Community Health Administration (CHA), Diploma in Health Administration, MD - Hospital Administration (HA), Diploma in Hospital Administration,
2. Community medicine; preventive and social medicine; public health: MD - Social and Preventive Medicine / Community Medicine, Diploma in Public Health (DPH), Diploma in Community Medicine (DCM).

(ii) Health (hospital) management / administration programs offered to medical and non-medical graduates through institutes (other than medical colleges) in:

1. Health management/ administration: PhD, Master of Health Administration (MHA), MBA, BBA, PGDPHM, Diploma
2. Hospital management/ administration: MBA, Master of Hospital Administration (MHA), BBA, MHM, PGDHM
3. Health and hospital management/ administration: PhD, MPhil, MBA, BBA, PGDHHM, Diploma
4. Master of Public Health (MPH).

PhD, Doctor of Philosophy; MPhil, Master of Philosophy; MBA, Master of Business Administration; PGDPHM, Post Graduate Diploma in Public Health Management; PGDHM, Postgraduate Diploma in Hospital Management; PGDHHM, PG Diploma in Hospital and Health Management; MHA, Master of Hospital Administration; MHM, Master in Hospital Management; BBA, Bachelor of Business Administration.

### Supply estimation

We estimated the annual supply of health management professionals (HMPs) graduating from the institutions using data from these six sources. We estimated the supply for (i) health (hospital) management/administration programs offered exclusively to medical graduates through medical colleges such as: MD—Community Health Administration (CHA), MD—Hospital Administration (HA), Diploma in Health Administration, and Diploma in Hospital Administration—from the data available on the website of Medical Council of India (MCI). Additionally, data for other courses offered by medical colleges such as: MD—Social and Preventive Medicine/Community Medicine, Diploma in Public Health (DPH) and Diploma in Community Medicine (DCM) was also obtained from the website of Medical Council of India (MCI).

For (ii) health (hospital) management/administration programs offered to medical and non-medical graduates through institutes (other than medical colleges), we identified the programs offering health management/administration, hospital management/administration, and health and hospital management/administration—as these programs impart training and offer jobs in health management. Similarly, we identified MPH programs being offered in the country, as health management is taught as a core module in these programs. Search for these institutions/programs was undertaken in Google search engine using keywords such as “health management programs,” “health administration programs,” “health management,” “health and hospital administration,” “MBA in health management,” “hospital management and administration,” “MPH” etc. However, we limited our search to programs offered in India and to collaborations between Indian and foreign institutions, if any. Additionally, the websites of the All India Council of Technical Education, University Grants Commission, and universities and institutions were also searched. In addition, education supplements of leading newspapers and education-based websites, including shiksha.com, targetstudy.com, getmyuni.com and career.webindia123.com, were searched. Related literature was reviewed through Google Scholar and PubMed and opinion from public health experts was taken in the field of health management education.

#### Assumptions for supply estimation

Based on our review and estimation for 2017, HMPs enter the Indian health system from two streams (six categories). Based on the differential nature of these programs, we considered following seat occupancy and placement percentages for HMPs graduating every year from these sources.

Additionally, on each supply capacity we have applied a three per cent migration rate for HMPs as around five per cent medical professionals migrate to developed countries (14). We have assumed the death rate of 3.1 per cent for this group based on the data on death rate as per Census 2010 (15).

- Medical colleges (programs in health management/ administration; hospital management/administration): It was assumed that seat occupancy would be 95 per cent for these programs. It was considered that 85 per cent graduates would work in health management/ administration services.
- Medical colleges (programs in community medicine; preventive and social medicine; public health): Like the former, we assumed that there would be 95 per cent seat occupancy and 20 per cent graduates would work in health management/administration services.
- Institutions offering programs in health management/ administration: We created three seat occupancy scenarios for the purpose of this work. We assumed a 70 per cent (low seat occupancy—best guess), 80 per cent (moderate seat occupancy—optimistic), and 90 per cent (high seat occupancy—aspirational)—scenario. Also, we assumed that 85 per cent graduates would work in health management/administration services.
- Institutions offering programs in hospital management/administration: Like the former, here also we assumed three scenarios: 70 per cent (low seat occupancy—best guess), 80 per cent (moderate seat occupancy—optimistic), and 90 per cent (high seat occupancy—aspirational). Due to overlapping job roles of the health/hospital management/ administration graduates, it was assumed that 85 per cent of the hospital management/ administration graduates would work in health management/administration services.
- Institutions offering programs in health and hospital management/administration: Here also we assumed three scenarios: 70 per cent (low seat occupancy—best guess), 80 per cent (moderate seat occupancy—optimistic), and 90 per cent (high seat occupancy—aspirational). It was assumed that 85 per cent of the health and hospital graduates would work in health management/administration services.
- Institutions offering Master of Public Health (MPH): Based on a recent study (16), we assumed a seat occupancy of 60 per cent (low seat occupancy—best guess), 68 per cent (moderate seat occupancy—optimistic), and 75 per cent (high seat occupancy—aspirational). It was assumed that only 30 per cent graduates would work in health management services post completion of MPH degree.

This helped us to enumerate the annual capacity for training HMPs in the year 2017. As stated by Sharma et al. that ~2122 health management seats were offered in 2011 (51 institutions) (3) while this number increased by 54 per cent to 3291 seats (75 institutions) in 2017–2018. For subsequent years, we assumed that the growth in the supply side for health management programs would be 50 per cent over the next decade for institutions offering health management programs. The numbers for the rest of the years was imputed for each intervening year from 2018 to 2029–for all three scenarios.

### Need estimation

The normative need for HMPs was calculated using “service target approach” for HMPs in the areas of practice, research and education. Need was accounted for trained health care managers/ administrators to work at block, district, and state level, national institutes (such as National Institute of Health and Family Welfare (NIHFW), National Health Systems Resource Centre (NHSRC), State Health Systems Resource Centers (SHSRCs) etc.), health based Non-Governmental Organizations (NGOs), academic/research organizations, corporate sector and international organizations. For calculating the normative need for these domains, the author replicated the methodology as used by authors in an earlier study (3) to calculate values for the year 2017.

Additionally, from the year 2017 to 2030, requirement for health management professionals was also calculated on the basis of benchmark analysis^1^. As per the “Potential Local Public Health Workforce Benchmarks” stated in National Association of County and City Health Officials (NACCHO) report there should be 2.97 health services managers per 100,000 population (17).

### Gap estimation

The number of HMPs currently in the workforce was inputted as 85% of current estimated need for HMPs using the service target approach for the base year 2017. For subsequent years, we estimated net HMPs in health workforce as the sum of number of HMPs in health workforce and HMPs produced annually minus HMPs exiting workforce through death (3.1 per cent) and migration (3 per cent).

#### Assumptions for need for public health cadre

Anticipating the setting up of Public Health Cadre in the country as outlined in the National Health Policy 2017, we assumed that-−1/3rd of the states will implement PH cadre by 2020, another 1/3rd by 2023 and all states by 2026. As per the Approach Paper on Public Health Cadre, we considered the following posts for health management professionals at different levels—one at state, two at district and one at each block level (18).

## Results

### Supply estimation

On the basis of information collected there are total 475 academic programs (offering health (hospital) management/administration) having enrolment capacity of 6963 seats collectively (2017). The details have been provided in Table 1.

**Table 1:** Supply of HMPs through medical colleges and institutes [offering health (hospital) management/administration].

**Sr. No**.	**Source**	**Education background as eligibility criteria**	**Name of program**	**Number of programs**	**Number of seats**
**MEDICAL COLLEGES OFFERING PROGRAMS IN:**					
1	Health management/ administration.; Hospital management/ administration;	Medical	MD - Community Health Administration (CHA), Diploma in Health Administration, MD - Hospital Administration (HA), Diploma in Hospital Administration,	11	53
2	Community medicine; preventive and social medicine; public health	Medical	MD - Social and Preventive Medicine / Community Medicine, Diploma in Public Health (DPH), Diploma in Community Medicine (DCM)	291	991
**INSTITUTES (OTHER THAN MEDICAL COLLEGES) OFFERING PROGRAMS IN:**					
3	Health management/ administration	Medical and non-medical	PhD, Master of Health Administration (MHA), MBA, BBA, PGDPHM, Diploma	45	2096
4	Hospital management/ administration	Medical and non-medical	MBA, Master of Hospital Administration (MHA), BBA, MHM, PGDHM	57	1454
5	Health and hospital management/ administration	Medical and non-medical	PhD, MPhil, MBA, BBA, PGDHHM, Diploma	27	1179
6	Master of Public Health (MPH)	Medical and non-medical	MPH	44	1190
TOTAL				475	6963

**Figure 1:**
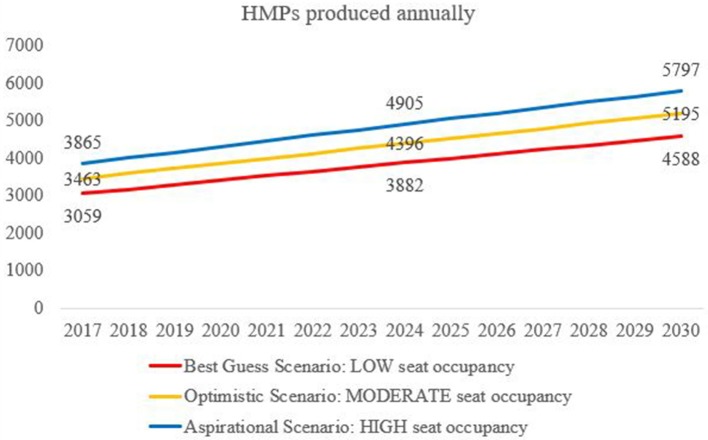
Health Management Professionals HMPs produced annually in Best Guess, Optimistic and Aspirational scenarios in India (2017 to 2026).

Thus, considering the differential seat occupancy for the year 2017, 2024, and 2030 it was observed that following number of HMPs are produced annually in the three scenarios (Figure 1). In case, the growth in the programs offering health management would continue to grow by 50 per cent by 2030 then it would increase from 3463 (2017) to 5195 by 2030 (in moderate scenario).

Considering the differential seat occupancy for the year 2017, 2024, and 2030 it was observed that following number of HMPs are produced annually from these two streams (six categories; Table 2).

**Table 2:** HMPs produced annually in best guess, optimistic and aspirational scenarios in India.

**Year**	**Scenarios—seat occupancy**	**Medical colleges**		**Other institutions**				**Total HMPs produced annually**
		**Health management/administration; Hospital management/administration**	**Community medicine; preventive and social medicine; public health**	**Health management/administration**	**Hospital management/administration**	**Health and hospital management/administration**	**Master of Public Health (MPH)**	
2017	Best Guess—Low	40	150	1193	829	673	174	3059
	Optimistic—Moderate	45	170	1351	939	762	197	3463
	Aspirational—High	50	189	1507	1047	850	220	3865
2024^*^	Best Guess—Low	50	190	1514	1052	854	221	3882
	Optimistic—Moderate	57	215	1714	1191	967	251	4396
	Aspirational—High	64	240	1913	1329	1079	280	4905
2030^*^	Best Guess—Low	60	225	1789	1243	1009	262	4588
	Optimistic—Moderate	68	255	2026	1408	1143	296	5195
	Aspirational—High	75	284	2261	1571	1275	330	5797

* *Forecasted estimates*.

### Need estimation through service target approach

Based on “service target approach” a normative need of around 11,304 HMPs was estimated for 2017, based on the number of positions available for HMPs in India. In the year 2010, as estimated by Sharma et al. there was an estimated requirement for 19,930 qualified health management professionals in the health sector (3). We updated the numbers for the year 2017, using similar methodology for our study. India with 36 states (29 states and 7 union territories), 640 districts and around 5,988 blocks will require a program manager at each level i.e., State Program Manager, District Program Manager and Block Program Manager respectively. Thus, ~6,664 trained professionals would be needed to serve these positions in the public sector.

Around a thousand consultants would be required in institutes like NIHFW, NHSRC, SHSRCs, etc. to work in their projects/departments. About 200 consultants/specialists will be needed at each state level across the country. More than 400 trained professionals would be employed across 90 large NGOs (19) in the country. Around 500 professionals would be needed across international organizations, while 1500 professionals would be necessary in academic/research organizations across the country. Similarly, around 1,000 professionals will be needed to work in Corporate Social Responsibility roles with corporate organizations. Thus, an estimated 11,304 HMPs are required to function in this capacity across the health sector

We assumed the following additional positions in health management with the setting up of the public health cadre—one at state, two at district and one at each block level.(18) At the country level we would need approximately 7,300 HMPs additionally in 2026 (i.e., 1 × 36 states + 2 × 640 districts + 1 × 5988 blocks). We assumed that - 1/3rd of the states will implement PH cadre by 2020, another 1/3rd by 2023 and all states by 2026. Thus, as per our estimates there will be a requirement of around 2435 HMPs by 2020, 5681 HMPs by 2024 and 7,304 HMPs by the year 2026.

### Need estimation through benchmark analysis

Assuming in India today, the number of HMPs in health workforce is around 9,608 (i.e., 85% of total 11,304 positions are occupied). Thus, if we calculate the number of health management professionals (HMPs) per one lakh population based on the current population of India i.e., 1.33 Billion (World Bank, Oct 2017) (20) then it comes out to be 0.72 HMP per 100,000 population. The normative need for HMPs was calculated on the basis of benchmark analysis in USA's scenarios of HMPs: population ratio. On the basis of NACCHO's “Potential Local Public Health Workforce Benchmarks” for Health services managers employed by local government there should be 2.97 HMPs per 100,000 population (17). As per this benchmark analysis, currently in the year 2017 there is a need of 39,774 HMPs in India to reach USA's 2.97:100,000 ratio. This would further grow up to 44,936 by the year 2030.

### Gap estimation

In the “moderate seat occupancy—optimistic scenario,” in the year 2017 there is a gap of 27,288 HMPs which is met by the year 2029. However, if the Public Health Cadre is instituted assuming-−1/3rd of the states will implement Public Health Cadre by 2020, another 1/3rd by 2023 and all states by 2026–then in the year 2030 around 7,304 HMPs; then this gap is not met by 2030.

**Figure 2:**
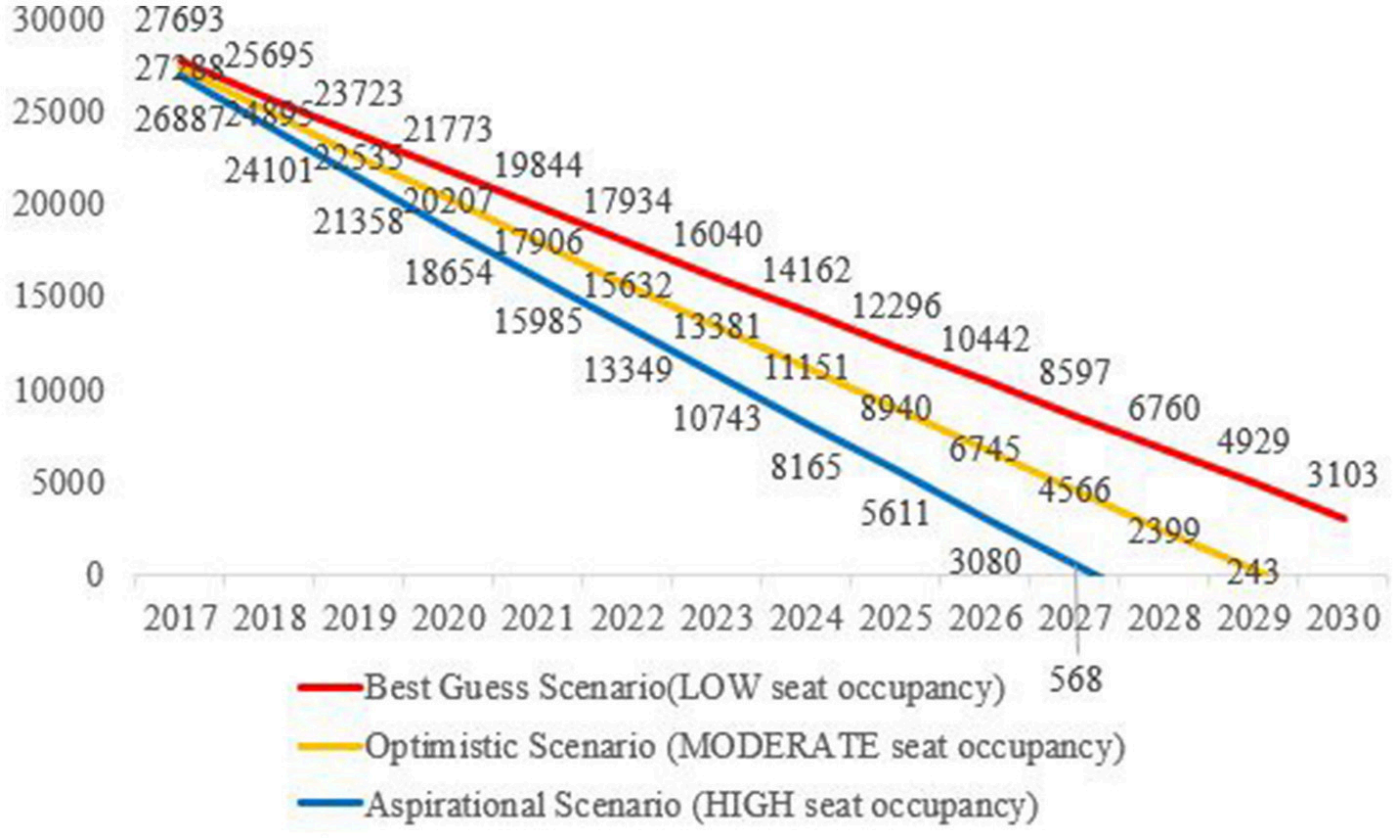
Gap in the number of HMPs calculated against a normative need of 2.97 HMPs per 100,000 population.

Similarly, in the “low seat occupancy—best guess scenario” in the year 2017 there is a gap of 27,693 PHPs which will not be met by 2030. By the year 2030 there is a gap of 10,407 HMPs in case the Public Health Cadre is instituted—with the requirement of 7,304 HMPs additionally. In “high seat occupancy—aspirational scenario” the gap is met by the year 2027 and in case Public Health Cadre is instituted then it is not met even by the year 2030. Figure 2 illustrates the three scenarios.

## Discussion

Globally, there is a felt need to reboot the health management education and practices. Health management practices traditionally focus on data collection and its processing for vital statistics, disease registries, and other surveillance-based resources (e.g., natality, morbidity, mortality, and some measure of environmental influences) for planning and operations of services (21, 22). In public heath, traditional health management practices includes the use of budgeting systems, financial performance measures and reports, and cost-control techniques for decisions which are unlikely to be sufficient for assessing how different health care activities and processes support a variety of health care policies goals. Whereas, present-day health management practices may include benchmarking, team-based performance measures and balanced information; which may support multiple goals by providing comprehensive information. This information may provide better control to the effectiveness of various management practices in supporting health care priorities both financial and non-financial; with a greater degree of functional structure coordination that helps in effective public health decision making (23).

It is also important to see how health management education and the role of health managers are patterned and consistent with other country's healthcare system. In the United States, fee-for-service, entrepreneurial dominated approach has resulted in a huge demand for additional health management education programs and managers (24). Thus, universities are attracted to establish health services administration programs (a term used in North America) as they require limited capital, continue to attract enrollment, and contribute to the “social good.” However, in the European countries, health systems in contrast provide universal access to care and strict, governmental fiscal control on healthcare expenditures (24).

Currently, there is a shortage of medical practitioners put strain on public health facilities in India (8). As per a India Brand Equity Foundation (IBEF) 2018 report, by 2030 India will need 2.07 million more doctors to achieve a 1:1000 doctor-to-population ratio (9). Thus, the answer perhaps lies in training of healthcare workers with non-medical background (creating HMPs) and relieving doctors of administrative and managerial responsibilities (9).

In India, health management programs are undersubscribed. These programs offered by medical colleges are regulated by Medical Council of India (MCI), whereas institutions training HMPs in India (includes Health, Hospital, Health, and Hospital Management and Administration programs)—are regulated by respective Universities and All India Council for Technical Education (AICTE). Similarly, MPH programs are regulated by their respective universities. It is pertinent to note that although there is an undersubscription in these programs, there was a period of rapid growth in the supply side in anticipation of a rise in the demand for health and hospital management expertise. The efforts of the NHM toward strengthening the management of public systems fueled this growth in the supply side. We anticipate that the creation of a public health cadre in the public system will continue to fuel this growth in the supply side in the coming decade. This optimism is also supported by the stated intent of the Government of India in the NHP 2017 document that prioritizes the set-up of a public health cadre in the country (10). The policy also advocates an appropriate career structure and recruitment policy to attract young and talented multidisciplinary professionals from—“sociology, economics, anthropology, nursing, hospital management, communications, etc. who have since undergone public health management training” (10). Thus, assuming a situation in which the Public Health Cadre is instituted in the country—with 1/3rd of the states implementing Public Health Cadre by 2020, another 1/3rd by 2023 and all states by 2026—then there will be a need of around 7,304 PHPs to work in Public Health Cadre by the year 2026.

As per WHO's Global Health Observatory, in the year 2011, China's health management and support workers density was 72 per 100 000 population (25). Currently in India, there are 0.72 HMPs per 100 000 population which is much lower than the stated benchmark of 2.97 HMPs per 100 000 population (17). However, in India we have not included support workers which includes “other categories of health systems personnel, which may include managers of health and personal-care services, health economists, health statisticians, health policy lawyers, medical records technicians, health information technicians, ambulance drivers, building maintenance staff, and other general management and support staff” —as per definition of WHO's Global Health Observatory (25).

As per a curricular review of MPH programs undertaken in the year 2015–health policy and management is covered in much greater depth in South Asian MPH programs (26). In a recent study undertaken by Pandav et al. health planning and management was described as a “core competency domain” for to be achieved by medical graduates (11). However, students enrolled in health management programs are not currently trained according to an explicitly stated, standardized competency framework that is tailored to the Indian context.

To meet the requirement of trained health professionals, globally, task-shifting as a practice is being used to help reduce the impact of insufficient health workers (27). It has been observed that non-physician clinicians, nurses can provide the same quality of primary care, for a set of common illnesses (28, 29). However, unlike medical skills, task shifting in public health management roles would be difficult. We do not advocate over-burdening of deficient clinical staff with additional management/ administration responsibilities in the health sector. We believe that such skills are additionally required in the health system for efficient functioning. We are not advocating that such skills can be provided only by non-medical management professionals. We visualize an important role for medical professionals trained in health management/administration since they bring a blend of medical and management skills. However, the specific requirement of how many such professionals would be required will have to be evolved through a wider consultative process.

Partnerships between institutions offering health management education and business schools could be very valuable (30). Additionally, MBA (general) graduates can also contribute to the pool of health management professionals. However, 70 per cent of the MBA (general) seats are vacant as the rush for these programs is now limited to premier institutes only (31). Those graduating MBA (general) programs in the country however, doesn't take up jobs in health sector as they don't find it lucrative enough for pursuing a career. Also, those hired in various national and international donor agencies, pharmaceutical sector, central, and state governments and the development partners—in the past have poor context of health.

The government would have to ensure and provide a conducive environment for sustaining a 50 per cent growth in the supply side of HMPs. Additionally, a growth in the demand side through setting up a public health cadre, additional job placements in the health and hospital management sector would also need to be created in the coming decade. Incase these changes are carried out in the future, we believe that this shortage for health management professionals will be met by 2,030.

## Author contributions

RT, HN, and SZ were involved in overall study design. RT conducted the literature review, collected the data. HN and RT analyzed the data. RT wrote the first draft of the manuscript which was reviewed and commented upon by HN and SZ who reviewed it critically for important intellectual content.

### Conflict of interest statement

The authors declare that the research was conducted in the absence of any commercial or financial relationships that could be construed as a potential conflict of interest.

## Footnotes

^1^simple benchmark ratios of required health management professionals' numbers (based on a benchmark) to appropriate populations.
